# Ileal prolapse through patent omphalomesenteric duct in a two year-old boy: a case report

**DOI:** 10.1186/s13256-024-04370-0

**Published:** 2024-02-05

**Authors:** Binyam Yohannes Wolde Hawariat, Amal Ousman Ali, Hana Abebe Gebreselassie

**Affiliations:** 1 Department of Surgery, St. Paul’s Hospital Millennium Medical Collage, Addis Ababa, Ethiopia; 2 Filtu General Hospital, Filtu, Somalia Ethiopia

**Keywords:** Patent vitelline duct, Omphalomesenteric duct, Prolapse, Umbilical discharge, Case report

## Abstract

**Background:**

Patent omphalomesenteric duct is one of the birth defects included in the spectrum of vitelline duct abnormalities. It is a rare anomaly with estimated prevalence of 0.13–0.2% in the general population. The most common presentation of patent vitelline duct is yellowish or mucoid type umbilical discharge which is usually noted in neonatal age or infancy. The main stay of diagnosis is clinical and outcome is favorable as long as timely surgical correction is offered. Here we present a 2 years old male child who presented with ileal prolapse through patent vitelline duct which is an exceptional mode of presentation of this pathology.

**Case presentation:**

2 years old Ethiopian male child who was noticed to have umbilical discharge since early infancy presented with protrusion of pinkish mass per the umbilicus of 4 h duration. He had no signs and symptoms of bowel obstruction. Abdominal examination revealed a prolapsed bowel which was viable via the umbilicus which was about 6 cm long. Otherwise, he had no abdominal tenderness or rigidity. He was explored with a smiley incision just above the umbilicus. The prolapsed bowel was reduced gently to the abdominal cavity. The tract of the Patent vitelline duct was identified and completely resected along with a wedge of ileum at its base. Primary repair of the ileal end where the tract was inserted was done in two layers and abdomen was closed in layers. The child had smooth post op course and was discharged on the 4th post-operative day.

**Conclusion:**

Prolapse of a bowel through the umbilicus is unusual presentation of a rare anomaly namely patent vitelline duct. This presentation warrants early surgical intervention before bowel ischemia issues. Hence, all clinicians dealing with children should be aware of this rare pathology so that urgent surgical management can be offered.

## Introduction

Several congenital and acquired disorders can present with umbilical discharge and mass in children. These include vitilline duct abnormalities, patent urachus, umbilical granuloma, umbilical polyp, omphalith, neoplasms and benign soft tissue masses. Among the congenital causes, vitilline duct abnormalities are the most commonly encountered disorders in the pediatrics population.

Vitelline duct or omphalomesenteric duct is a structure connecting the mid gut and the yolk sac in the early weeks of gestation. The anomalies that arise from this structure occur due to its failure of obliteration by the 5th to 9th weeks of fetal life. The prevalence of vitelline duct anomalies in the general population is said to be 2–3% [1, 2].

A spectrum of anomalies can arise from the vitelline duct depending on the site and the extent of its persistence. These include patent vitelline duct, Meckel’s diverticulum, cyst, sinus and fibrous cord. Meckel’s diverticulum is the most common vitelline duct anomaly. Patent vitelline duct is reported in about 15% of all the vitello-intestinal duct anomalies [3].

Although Meckel’s diverticulum is the most common vitelline duct anomaly, a patent vitelline duct is the most common symptomatic presentation in low income countries [4]. It usually presents during early infancy with fecal discharge through the umbilicus but it is also reported in older children and even adults [3, 5, 6]. One of the rare presentations of patent vitelline duct is prolapse of small bowel to the exterior through its opening on the umbilical side. Here we present a 2 years old male child who presented with prolapse of ileum via patent vitelline duct.

## Case summary

**Fig. 1:**
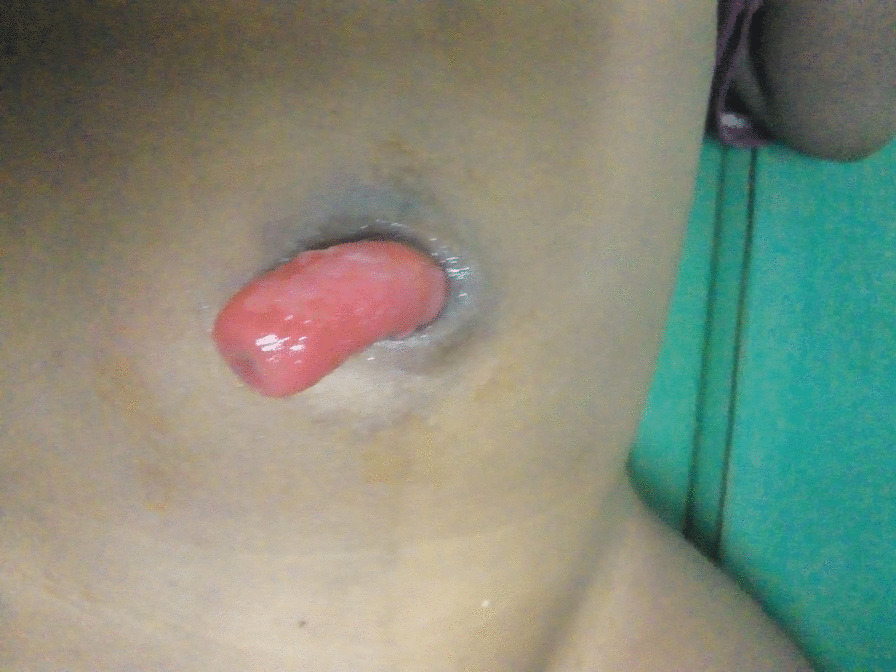
Preoperative appearance of prolapsed ileum via patent vitelline duct

A 2 years old Ethiopian male child presented with protruding mass per umbilicus of 4 h duration. He was noticed to have umbilical discharge which was yellowish and at times mucoid since infancy by the parents. He has no vomiting, abdominal cramp, obstipation or abdominal distension at the time of presentation. He had no gross dysmorphic feature and there was no known associated congenital anomaly. He had no previous visit to a health facility and no known medical illness. On examination, he was comfortable with normal vital signs. He had normal weight and height for his age. Abdominal examination revealed a prolapsed bowel which was viable via the umbilicus which was about 6 cm long (Fig. 1). Otherwise, he had no abdominal tenderness or rigidity. He was investigated with complete blood count and all parameters were within the normal limit.

**Fig. 2:**
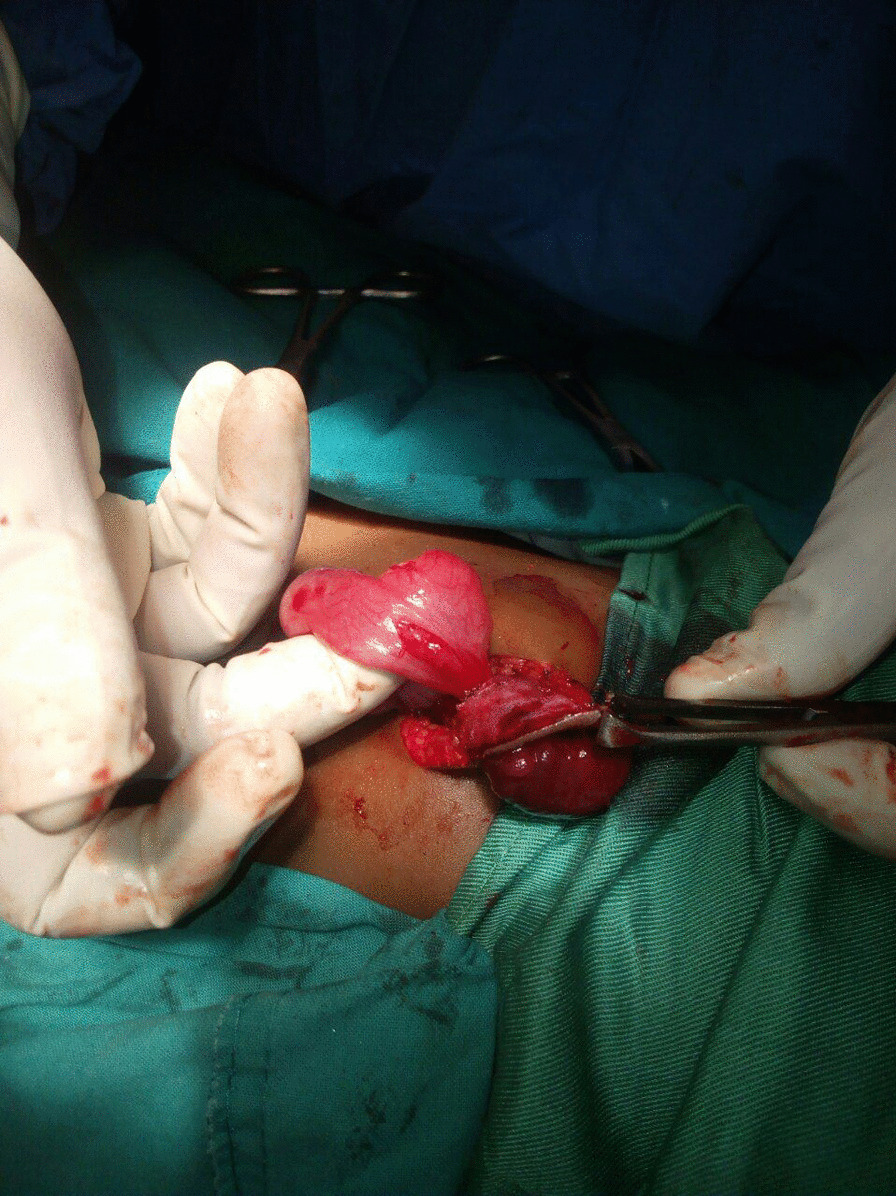
Intra operative picture showing the ileal segment protruding via patent vitelline duct

With a diagnosis of patent vitelline duct with ileal prolapse, consent was obtained from the parents and he was taken to the operation theater the same day for exploration. Manual reduction of the prolapsed bowel was tried first but it was difficult. Hence, an inverted smiley incision was made just above the umbilicus and peritoneal cavity was entered. The prolapsed bowel which was edematous at this point with the manipulation was reduced gently into the abdominal cavity. The tract of the Patent vitelline duct was identified and completely resected along with a wedge of ileum at its base. Primary repair of the ileal end where the tract was inserted was done in two layers and abdomen was closed in layers (Fig. 2). Post operatively, the child was put on antibiotics, analgesics and maintenance fluid. Subsequently, he had smooth in hospital course and discharged on the 4th post-operative day. He was seen 1 month after the surgery at the clinic and he was thriving well with no complications.

## Discussion

Patent vitelline duct occurs when the entire omphalomesenteric duct fails to obliterate at the 9th to 10th week of gestation. Hence, part of the mid gut to which this duct is inserted to will be in communication with the umbilicus. Other terms which are used to describe this anomaly include omphalloileal fistula and umbilico- intestinal fistula [2].

The prevalence of vitelline duct anomalies in the general population is said to be 2–3% and out of this, 15% is accounted by patent vitelline duct [1, 2]. Hence, the prevalence of patent vitelline duct is 0.13–0.2% which shows that it is one of the rare anomalies. The male prevalence of this disorder can be seen in most of the publications on this subject as it was in our case [2, 4].

A number of differential diagnoses can be considered in a child who presented with umbilical discharge and mass. These include vitilline duct abnormalities, patent urachus, umbilical granuloma, umbilical polyp, omphalith, neoplasms and benign soft tissue masses. The presence of yellowish and\or mucoid discharge since early infantile age and the acute presentation with protrusion of a bowel segment via the umbilicus in our patient makes the likely diagnosis to be patent vitilline duct.

The most common age of presentation of patent vitelline duct as described in various literature is during neonatal age and infancy [1, 2, 4, 7–10]. Even though, our patient was noticed to have umbilical discharge since early infancy, his parents didn’t seek medical attention until prolapse of bowel through patent vitelline duct occurred.

Children with patent vitelline duct can present with umbilical discharge of fecal or mucoid matter or they can also present with intestinal obstruction. In a review done by Rajandra, most of the patients (54.1%) presented with signs of bowel obstruction and among these patients, 92% had bowel prolapse through the patent vitelline duct. On the other hand, the rest 45.9% of his patients came with simple umbilical discharge without bowel obstruction [2]. The child in our case presented with bowel prolapse but no signs of obstruction. This may be due to the early presentation of this patient to the hospital from the time of prolapse.

Surgery is the mainstay of management for patent vitelline duct as it has to be fully excised. The commonly used incision to approach a patent vitelline duct with bowel prolapse is smiley periumbilical incision even though a paramedian and midline incision have been described [8, 10]. The most common part of the mid gut to which patent umbilical duct was inserted to was the ileum [1, 4, 7–10]. Other parts of bowel that had connection with this duct and described on literature were the appendix and the ascending colon [4].

As to the surgical management, various options were used to handle the bowel after full excision of the duct. In cases where part of the bowel was ischemic, resection and end to end anastomosis and rarely resection with ileostomy was done [2]. On the other hand, in patients with viable bowel resection of the bowel receiving the fistula tract with end to end anastomosis is one option. The other option is to do wedge resection of the bowel followed by primary repair. We used the last procedure in our patient.

The outcome of patients who present with bowel prolapse through patent vitelline duct as described on the published literature is favorable especially in those who had early presentation. Three deaths were reported in the publications we reviewed and all of these deaths were ascribed to sepsis secondary to peritonitis which followed anastomotic leak.

## Conclusion

Prolapse of a bowel through the umbilicus is unusual presentation of a rare anomaly namely patent vitelline duct. This presentation warrants early surgical intervention before bowel ischemia issues. Hence, all clinicians dealing with children should be aware of this rare pathology so that urgent surgical management can be offered.

## Data Availability

Any further data that might be needed can be obtained from the corresponding author upon request.
